# Osteochondroma of the atlas vertebra causing high grade spinal canal stenosis: a rare case report

**DOI:** 10.1259/bjrcr.20230010

**Published:** 2023-04-27

**Authors:** Shweta Aggarwal, Rajat Sachdeva

**Affiliations:** 1 Department of Radio-Diagnosis, Rama Medical College, Hospital and Research Centre, Hapur, India

## Abstract

Osteochondromas are the most common primary benign bone tumors which can be either solitary or multiple in the form of hereditary multiple exostosis (HME). Osteochondromas are located frequently in the long bones and rarely involve the spine. Cervical spine remains the most common site for spinal osteochondroma. However, majority of the cases are neurologically asymptomatic as most of them are slow growing with growth directed outside the spinal canal. In this case report, we describe a rare case of solitary osteochondroma arising from C1 vertebra (atlas) resulting in serious neurological complications, ultimately necessitating surgical intervention.

## Clinical Presentation

A 25-year-old male patient was referred to Neurology OPD with 4 year long history of weakness, numbness and loss of movements in right upper limb which gradually progressed to involve left upper limb and bilateral lower limbs. Later in course of his symptoms, he started complaining of bowel and bladder dysfunction.

He had a history of fall during sleep 1 year back followed by loss of consciousness. There was no history of fever or seizures.

He had been taking ayurvedic treatment for his illness which showed no signs of improvement.

Initial workup showed vitals within normal limits. On examination, Grade II bedsores were detected. Extensor planter reflexes and Grade 3+ knee and ankle reflexes were noted bilaterally. Ultrasound of whole abdomen revealed thickened and irregular walls of urinary bladder with low level internal echoes & debris—consistent with cystitis.

Plain radiograph of the cervical spine which was done at a primary healthcare facility revealed a bony lesion in posterior spinal column at the level of C1 vertebra. Subsequently, patient was referred to Rama Super-speciality Hospital for further investigations which included CT and MRI of the cervical spine.

## Imaging findings

**Figure 1.**
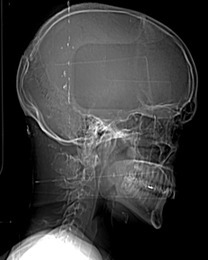
CT scout image shows a well-defined osseous outgrowth in posterior column of upper cervical spine at C1/C2 vertebral level.

**Figure 2.**
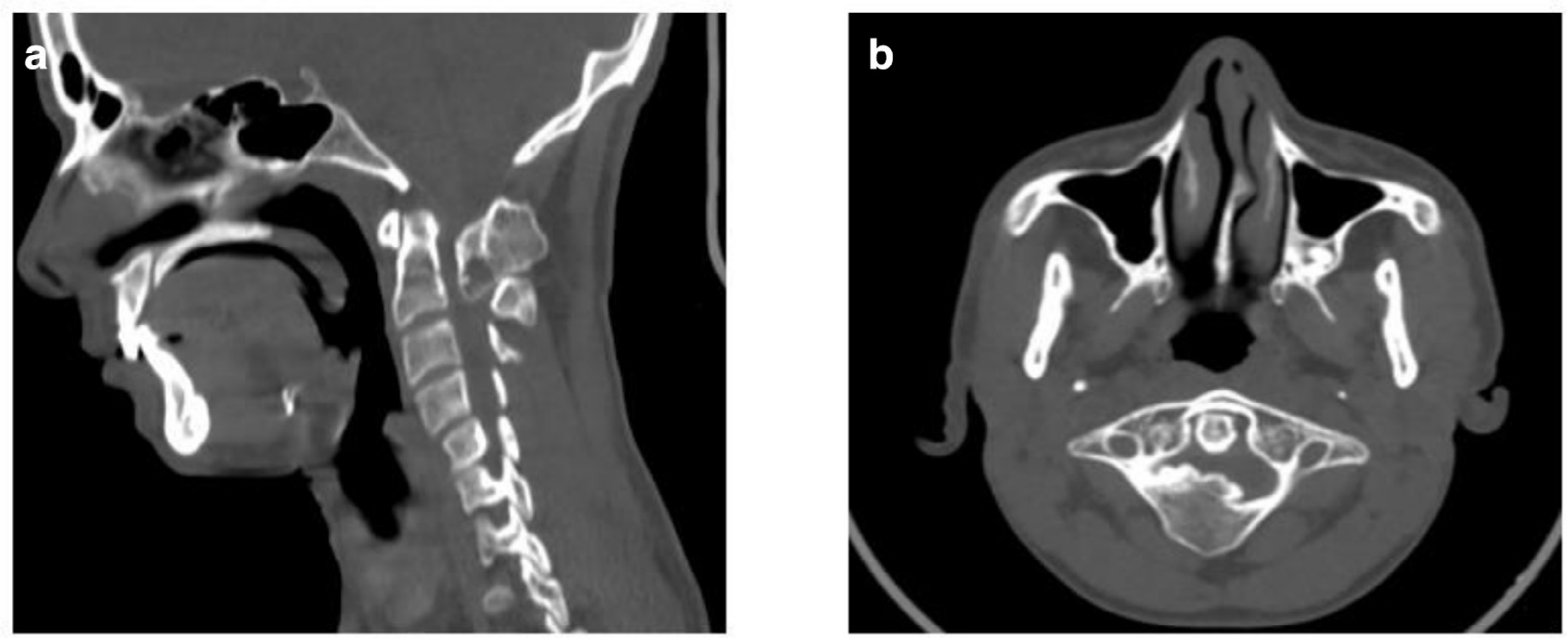
(a, b) Sagittal and axial CT of cervical spine in bone window at the level of C1 vertebra shows a sclerotic outgrowth arising from posterior arch of atlas and protruding into the spinal canal. The lesion shows no clear demarcation with the surrounding normal bone. Lesser ossified anteroinferior portion of lesion is better seen in sagittal view.

CT topogram image of the cervical spine showed a well defined osseous lesion in upper posterior cervical region (Figure 1). Non-contrast CT scan (NCCT) with multiplaner reconstructions revealed a well-defined sclerotic expansile lesion arising from the right half of posterior arch of C1 vertebra, extending through the midline to involve medial aspect of hemi left posterior arch measuring ~2.4 x 2.9 x 2.5 cm (AP x TR x CC). Bony trabeculae seen within the lesion appeared to be in continuation with medullary cavity of rest of the normal bone (Figure 2a and b). No evidence of surrounding periosteal reaction/soft tissue component/ signs of bony destruction was present.

**Figure 3.**
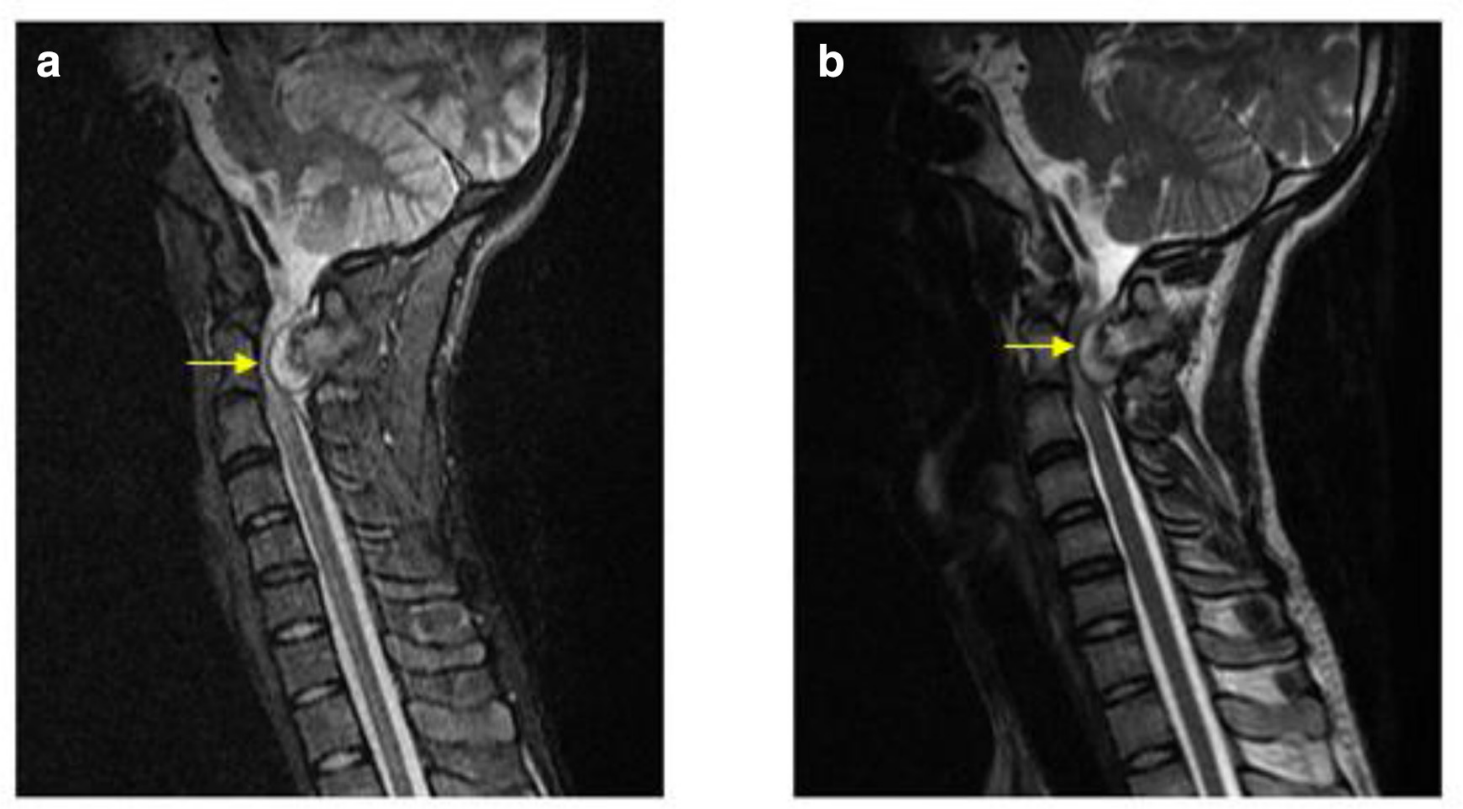
(a, b) Sagittal STIR & *T*_2_ weighted MRI shows a hyperintense cartilaginous cap along the anteroinferior margin of the bony growth. Note the mass effect on the spinal cord. STIR, short tau inversion recovery.

Further, pre- and post-contrast MRI of cervical spine was done to assess for status of the spinal cord and to determine the signal characteristics of the lesion. The lesion appeared isointense to the adjacent bone on all MR sequences. Additionally, a small T2/STIR hyperintense component was noted along its anteroinferior margin—likely representing a cartilaginous cap, measuring approximately 5.2 mm in thickness (Figure 3a and b).

**Figure 4.**
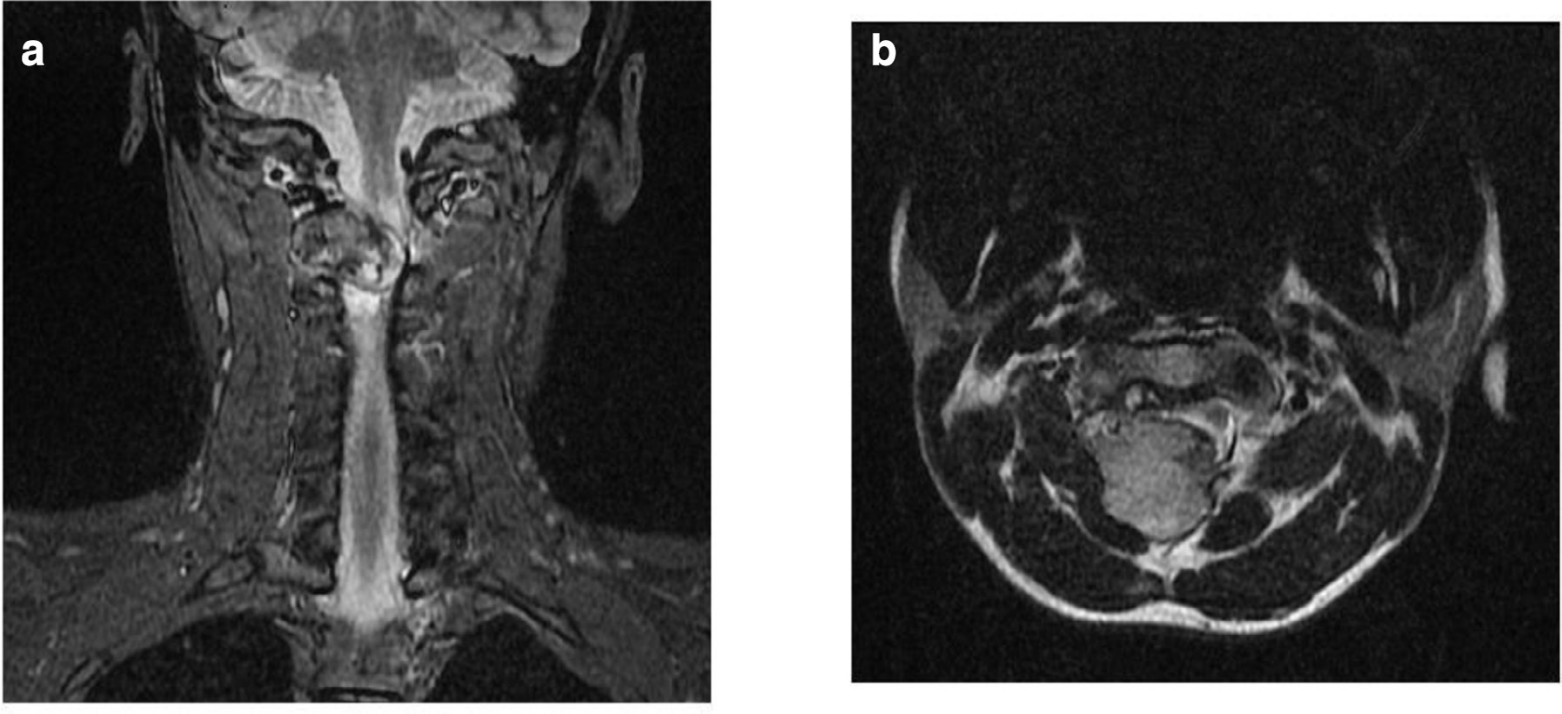
(a, b) Coronal STIR and axial T2 MR sequences show gross displacement of spinal cord anteriorly and towards left at C1-C2 level with near total canal stenosis. STIR, short tau inversion recovery.

This osseous growth caused marked extrinsic compression over the posterior dural sac resulting in displacement of the spinal cord towards left side at C1-C2 level and high grade spinal canal stenosis (AP canal diameter measures approximately 2.5 mm) (Figure 4a and b).

**Figure 5.**
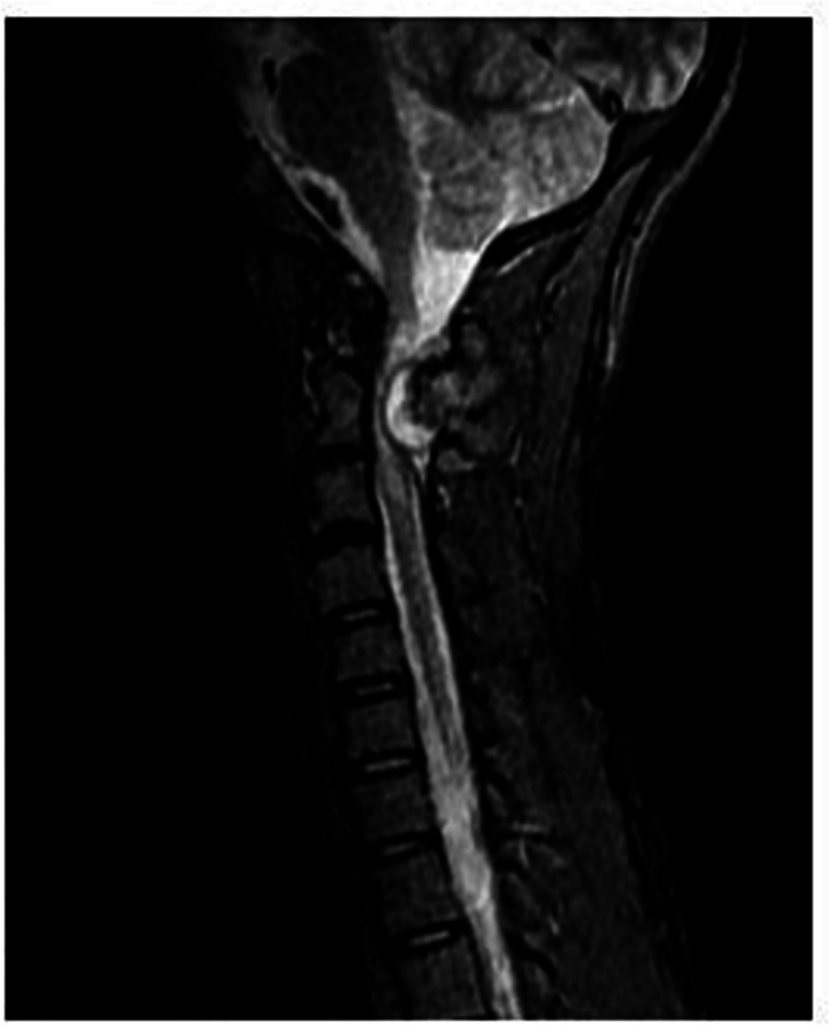
Sagittal STIR image shows hyperintense intramedullary signal extending from C1 to C2 level indicating compressive myelopathy changes within the cervical cord. STIR, short tau inversion recovery.

**Figure 6.**
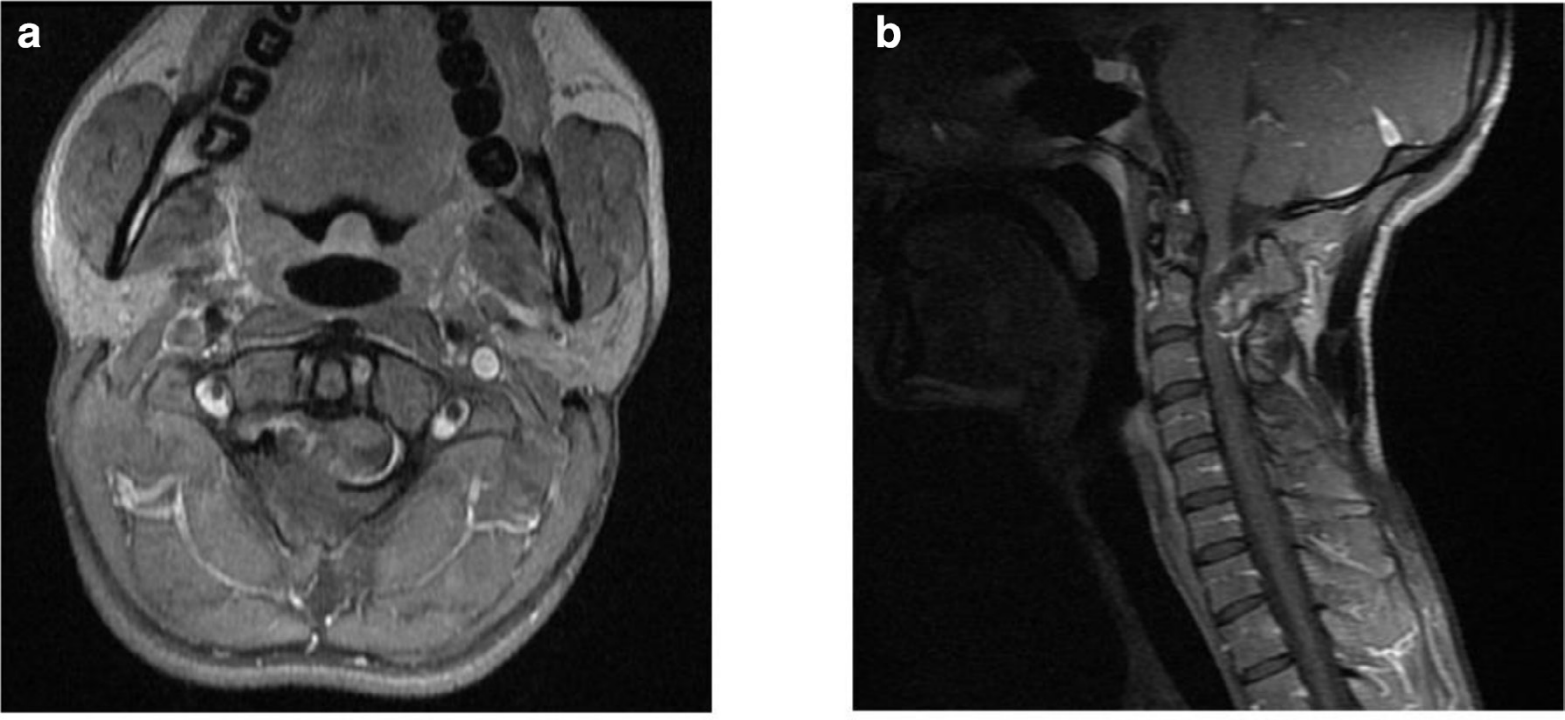
(a, b) Contrast *T*_1_ weighted axial and sagittal MR sequences do not reveal enhancement within the lesion.

Intramedullary T2/STIR hyperintense signal was also seen at the level of canal stenosis extending upto a length of 2.5 cm—suggestive of cord myelomalacia due to compressive myelopathy (Figure 5). On post-contrast study, no obvious enhancement of the lesion was detected (Figure 6a and b).

## Discussion

Osteochondromas are the most common primary benign bone tumors contributing to 36% of all benign bone tumors.^1^ They can either be solitary (90% of cases) or multiple as a part of inherited syndrome called hereditary multiple exostosis (HME) (10% of cases).^2^ Osteochondromas are located frequently in the long bones and rarely involve the spine, representing for only 4–7% of primary benign spinal tumors^3^ and less than 3% of all osteochondromas. Several studies have demonstrated that solitary osteochondromas are more common in the spine than osteochondroma associated with HME.^4^ HME is associated with higher incidence of spinal involvement (3% of cases) and neurological complications than solitary osteochondromas.^5^ Solitary vertebral osteochondromas with spinal cord compression are extremely rare.^6^

Osteochondroma is a disease of the growing skeleton and can either be sessile or pedunculated. Therefore, it commonly presents in young patients and its growth usually arrests after puberty with closure of the epiphysis.^7^ Most of them are asymptomatic and are detected incidentally. Symptomatic lesions usually occur in younger patients, with 75–80% of such cases being discovered before the age of 20 years.^8^ The early diagnosis in HME is attributed to multiplicity of lesions and associated deformities in contrast to the relatively later diagnosis of solitary osteochondromas.^9^

In spinal osteochondromas in the setting of HME, thoracic and lumbar spine are more commonly affected, while the solitary type has higher predilection for the cervical spine particularly the atlantoaxial region.^10^ In vertebral bodies, the secondary ossification centers are located within the endplates, which fuse during adolescence. These ossification centers appear at an earlier age and faster in the cervicothoracic spine compared to lumbar spine.^11^ The more rapid the ossification of these centers, higher the chances of development of aberrant cartilage, which explains why osteochondromas are more common in the cervical spine. Another possible reason for involvement of cervical spine is greater mobility and stress in this region. Lamina is the origin of most spinal osteochondromas.

In our case, a solitary and sessile vertebral osteochondroma originating from the posterior arch (posterior column) of atlas and protruding into the middle column has caused severe spinal cord compression, thereby making it a rare occurrence. The continuity between cortex and medulla of the lesion with that of the host bone remains the most important and pathognomic feature in the diagnosis of osteochondroma.^2,10^ This is best demonstrated on CT scan, as in this case.

MRI shows the medullary and cortical components of the lesion which appear isointense as the normal bone. MRI often shows yellow marrow signal (T1/T2 hyperintense) in the central aspect of the lesion. The maximum thickness of the cartilage cap is important and MRI is more accurate than CT scan in this regard. The cartilage cap has low to intermediate signal on *T*_1_ weighted images and high signal on *T*_2_ weighted images and STIR. Cartilage caps upto 2 cm and calcified caps are considered features of benignity whereas caps above 2 cm are considered malignant.^12^ In the presented case, maximum thickness of the cap was 5.2 mm. Other concerning signs of malignant transformation include invasive bony destruction, associated soft tissue component and growth after skeletal maturity. MRI is also important to assess any nerve root or spinal cord compression in clinically symptomatic patients.

In a review study done for 11 cases of adult cervical spine osteochondromas, it was found that majority of the tumors originated from posterior elements; however, cord compression was found in only one case—thereby making it a rare occurrence.^13^

Treatment for symptomatic spinal osteochondromas causing neurological compromise warrants surgical management which includes *in situ* marginal or wide excision, via a posterior, anterior, or combined approach, with or without instrumentation. In some cases, a need for cord decompression along with instrumented stabilization with or without fusion may be required.

In our case, excision of tumor with bilateral C1 arches and cord decompression was performed using posterior approach. Additional anterior stabilization was done by occipito-C3-C4 fusion. Histopathological analysis of the excised lesion revealed tissue undergoing enchondral ossification with underlying bony trabeculae enclosing marrow spaces; thereby confirming the radiological diagnosis. Patient was discharged in a neurologically stable condition and was followed-up weekly on out-patient basis. There was significant improvement in the muscle power and gradual restoration of limb movements with aided physiotherapy.

## Differential Diagnosis

Due to characteristic imaging appearance, there are limited differentials for spinal osteochondroma; however, low-grade chondrosarcoma may show a similar appearance. Often used interchangeably, the term Exostoses should be preserved for bony outgrowths lacking the cartilaginous cap.

## Learning Points

- Spinal osteochondromas are a rare entity, and may lead to serious neurological complications when associated with cord/nerve root compression.
- MR imaging is superior to CT for assessment of the relationship of osteochondroma to the surrounding structures for deciding the surgical approach.
- Complete resection of the characteristic cartilage cap seen with these tumors is of utmost significance during surgical excision as incomplete resection is associated with higher risk of recurrence.
